# Comparing Aortic Valve Replacement through Right Anterolateral Thoracotomy with Median Sternotomy

**Published:** 2013-09-01

**Authors:** Abdul Gani Ahangar, Aakib Hamid Charag, Mohd Lateef Wani, Farooq Ahmad Ganie, Shyam Singh, Syed Asrar Ahmad Qadri, Zameer Ahmad Shah

**Affiliations:** 1 Department of Cardiovascular and Thoracic Surgery, Soura, Kashmir, India

**Keywords:** Thoracotomy, Sternotomy, Aortic Valve

## Abstract

**Background:**

Aortic Valve Replacement (AVR) is usually done through median sternotomy. The present study aimed to compare the right anterolateral thoracotomy and median sternotomy approaches for AVR.

**Materials and Methods:**

The present prospective study was conducted on 60 patients who had aortic valve disease and were subjected to AVR. Thirty patients underwent aortic valve replacement via right anterolateral thoracotomy (study group) and thirty patients via median sternotomy (control group). Statistical analysis was done using Mann Whitney U test and Fischer’s Exact test. Statistical Package SPSS ­17 was used for data analysis.

**Results:**

The mean length of the incision was 18.7±1.8 cm in the patients who had undergone AVR through median sternotomy, while 7.8±0.9 cm in the study group patients. Besides, the mean bypass time was 121.8±18.6 minutes for the patients who had undergone AVR through median sternotomy, while 122.1±20.8 minutes for the study group. In addition, the mean aortic cross clamp time was 67.7±13.4 minutes for the patients who had undergone AVR through median sternotomy, while 68.0±8.9 minutes for the study group. The mean operating time was 181.6±31.5 minutes for the patients who had undergone AVR through median sternotomy, while 190.8±29.8 minutes for the study group. Patient satisfaction with respect to cosmesis was higher in the study group. Only 50% of the patients who had undergone AVR through median sternotomy in comparison to 73.3% of those in the study group were satisfied with the cosmesis.

**Conclusions:**

The right anterolateral thoracotomy approach for aortic valve replacement proved to be easy to perform whilst maintaining the maximum security for the patients. Besides its better cosmetic result especially in female patients, this approach proved to have several advantages.

## 1. Background

Aortic Valve Replacement (AVR) can mostly be performed through either a median sternotomy or a right anterolateral thoracotomy. Of course, many alternative approaches, such as partial T or L sternotomy through the third or fourth intercostal space, reversed T sternotomy, transverse sternotomy, parasternotomy with excision of two or more costal cartilages, and various types of anterolateral mini­thoracotomies, have been described that lessen the damage to the thoracic cavity. The conventional median sternotomy may cause significant surgical trauma and morbidity. Moreover, obese patients and diabetics are particularly prone to sternal infection and instability. Deep Sternal Wound Infection (DSWI) is a significant cause of morbidity and mortality following median sternotomy in cardiac surgery patients. It may follow sternal dehiscence which usually manifests 3­5 days postoperatively. Superficial Wound Infection (SWI) is considerably more common than DSWI, with the incidence of 3-10% among cardiac surgery patients. Furthermore, hypertrophic scarring is common with midline incisions, and keloid scars are especially likely to develop in the patients of African descent. Scar stretching is also a known complication of median sternotomy. The minimally invasive thoracotomy approach described here may have several advantages over median sternotomy. These potential advantages include better cosmesis, avoidance of prior sternotomy incision, quicker return to normal activity, less incisional pain, less blood loss, and less wound infection. It should be mentioned that particularly cosmetic results are excellent in women (1).

## 2. Materials and Methods

The present prospective study was carried out in the Department of Cardiovascular and Thoracic Surgery at Sher­i­Kashmir Institute of Medical Sciences, Srinagar. The study was conducted on the patients who had undergone AVR from September 2010 to August 2012. A detailed clinical examination was done on every patient once admitted in the hospital. Investigations for every patient included complete hemogram, blood grouping, kidney function test, liver function test, serum electrolytes, electrocardiograph, chest radiograph, coagulogram, and echocardiography.

An equal number of patients were randomly allocated to each group by computer generated numbers. The two groups were matched with respect to the different parameters as already described. The follow up information was obtained prospectively by following these patients in the outpatient clinic. The study population included 60 patients who had aortic valve disease and were subjected to AVR. Thirty patients underwent AVR via right anterolateral thoracotomy (study group) and thirty patients via median sternotomy (control group). High risk patients (ASA 3 or 4), patients with coagulation disorders, previous cardiac surgery, associated coronary artery disease, associated mitral valve disease requiring surgical intervention, and those who had not signed written informed consents were excluded from the study. The same general anaesthetic techniques with routine arterial and venous monitoring were utilized for both groups. In the study group, the patient was positioned supine, withhe right side chelevated to approximately 30 degrees by a roll beneath the shoulder. The right arm was also adequately padded and suspended over the head. The patient was then drapped in the usual fashion with exposure of the sternum and right side chest up to the posterior axillary line. Afterwards, an incision was made in the right sub­mammary fold starting at 3­5cm from the lateral border of the sternum. The breast tissue was gently mobilized and the right side chest cavity was entered through the 3rd intercostal space. The third costal cartilage was disarticulated from the sternochondral junction. A chest retractor was placed and opened gradually so as not to break any ribs. The right lung was compressed with a wet lap to expose the pericardial sac. Then the pericardial sac was entered through an incision 2­3cm anterior and parallel to the phrenic nerve extending from the diaphragm to the aortic reflection. Cannulation of SVC, IVC, and ascending aorta was done through the same access. Finally, the patient was put on the cardio­pulmonary bypass and AVR was performed.

For the control group, on the other hand, the patients were placed in supine position and AVR was performed through the standard median sternotomy. The patients were electively ventilated for some hours after the completion of the surgery. Post extubation patients were shifted from ICU after completely assessing the general condition and hemodynamics of the patients along with baseline investigations and blood gases. Intravenous morphine (3mg q6h) was used as analgesic for all the patients. In addition, oral anticoagulation was started on the 2nd postoperative day with acenocoumarol to maintain an INR of 2.0­2.5. Intravenous antibiotics, a combination of ceftriaxone/sulbactam, and amikacin were also administered during the hospital stay and changed as and when needed as per cultural sensitivity.

### 2.1. Statistical Analysis

The results were presented as mean±standard deviation and percentages. The study data did not follow the normal distribution which compelled us to rely upon the non­parametric tests, such as Mann Whitney U test, in order to determine the significant differences.

Moreover, Fischer’s exact test was to determine the association between the variables. Statistical Package SPSS­17 was used for data analysis.

## 3. Results

The mean age of the patients who underwent AVR through median sternotomy and right anterolateral thoracotomy was 36.6±6.7 and 38.5±10.6 years, respectively. Among the patients who underwent AVR through median sternotomy, 43.33% were male, whereas 56.66% were female. On the other hand, 26.66% and 73.3% of the patients who underwent AVR through right anterolateral thoracotomy were male and female, respectively. The majority of the patients who underwent AVR through median sternotomy had NYHA Class III (93.33%). However, only 6.66% of the patients had NYHA Class IV and none of the patients had NYHA Class II. In the study group, 46.7% of the patients had NYHA Class III, whereas 33.3% and 20% of the patients had NYHA Class IV and II, respectively. Among the patients who underwent AVR through median sternotomy, 56.7% had ejection fraction between 40%­50%, 36.7% had ejection fraction >50%, and 6.6% had ejection fraction <40%. In the study group, on the other hand, 60% of the patients had ejection fraction between 40%­50%, 33.3% had ejection fraction >50%, and 6.7% had ejection fraction <40%. The mean length of the incision was 18.7 cm in the patients who underwent AVR through median sternotomy, while 7.8 cm for the patients in the study group. In addition, the mean bypass time was 121.8 minutes for the patients who underwent AVR through median sternotomy, whereas 122.1 minutes for the study group. The mean aortic cross clamp time was 67.7 minutes for the patients who underwent AVR through median sternotomy, while 68.0 minutes for the study group. Besides, the mean operating time was 181.6 minutes for the patients who underwent AVR through median sternotomy, whereas 190.8 minutes for the study group. The patients who underwent AVR through median sternotomy stayed in ICU for a mean duration of 29.7 hours, whereas those who underwent AVR through right anterolateral thoracotomy had a mean ICU stay of 26.1 hours. Numerical Rating Scale (NRS) was used for assessment of pain post­operatively. On the scale of 0­10, the patients were instructed to point out the intensity of pain during the last 24 hours. This assessment was done at 24, 48, and 72 hours after extubation. The average pain score was higher in the patients who underwent AVR through median sternotomy at all the three occasions compared to those in the study group. The results revealed a significant difference between the two groups in this regard at all the three occasions, with median sternotomy approach being more painful.

The patients who underwent AVR through median sternotomy stayed for an average period of 8.0 days post­operatively in the hospital, whereas the average post­operative hospital stay was 6.9 days for the patients who underwent AVR through right anterolateral thoracotomy. Moreover, 13.33% of the patients who underwent AVR through median sternotomy developed wound infection, while wound infection was detected in only 6.7% of the patients in the study group. Nevertheless, none of the patients in either group had wound dehiscence. Furthermore, 43.33% of the patients who underwent AVR through median sternotomy in comparison to 6.7% of the patients in the study group developed scar complications. Patient satisfaction with respect to cosmesis was higher in the study group. Only 50% of the patients who underwent AVR through median sternotomy compared to 73.3% of the study group patients were satisfied with the cosmesis (Table 1). 

**Table 1:** Comparision of Different Parameters in the Two Groups

Parameter		Right Anterolateral Thoracotomy (n=30) (mean ±SD)	Median Sternotomy (n=30) (mean ±SD)	*P *Value
**Age**		38.5±10.6	36.6±6.7	0.585
**Length of Incision (cm)**		7.8 ±0.9	18.7 ±1.8	< 0.001
**MBT (mins)**		122.1 ±20.8	121.8 ±18.6	0.983
**ACCT (mins)**		68.0 ±8.9	67.7 ±13.4	0.389
**Operating Time (mins)**		190.8 ±29.8	181.6 ±31.5	0.965
**ICU Stay (hours)**		26.1 ±7.3	29.7 ±12.9	0.394
**Post-op Hospital Stay (days)**		6.9 ±1.0	8.0 ±1.4	0.013
**Average Pain Score**		4.2 ±0.6	5.4 ±0.6	< 0.001
**Wound infection N(%)**		2 (6.7)	4 (14.3)	0.598
**Wound dehiscence N(%)**		0 (0)	0 (0)	-
**Scar Complication N(%)**		2 (6.7)	12 (42.9)	0.035
**Patient Satisfaction with cosmesis N(%)**	Yes	22 (73.3)	14 (50.0)	0.433
	No	2 (6.7)	7 (21.4)	
	Indifferent	6 (20.0)	8 (28.6)	

## 4. Discussion

Median sternotomy, which is generally used as a standard access for aortic valve operations, has a significant risk of postoperative infection and dehiscence. Moreover, the resulting large scar is a poor cosmetic result that may have adverse psychological consequences, especially on young women (2). These difficulties may be avoided by the use of a less invasive approach consisting of a limited right anterolateral thoracotomy with standard cannulation. We studied whether such complications can be avoided by choosing right anterolateral thoracotomy, simultaneously comparing the procedure with median sternotomy for certain intraoperative and postoperative parameters.

We studied a total of 60 patients out of which, 30 underwent AVR through median sternotomy and 30 through right anterolateral thoracotomy. In our study, the patients in the two groups were similar with respect to their mean age. The mean age of the patients was 36.6±6.7 and 38.5±10.6 years in the median sternotomy and the study group, respectively. Females formed the majority of the patients in our study (57.1% in the median sternotomy group and 73.3% in the study group). Minale C and colleagues performed aortic valve replacement in 50 patients with the mean age of 68+8.3 years who were relatively older compared to our patients (3).

The mean length of incision was 18.7±1.8 cm in the patients who underwent AVR through median sternotomy, whereas 7.8±0.9 cm in the study group (P<0.001). Federico J. Benetti and colleagues (4) in 1997 performed AVR in two patients via right anterolateral thoracotomy. A 6 cm incision was made in the third intercostal space in one patient and a 7 cm one in the other. In addition, Carmine Minale and colleagues (3) in 1998 performed AVR via a mini­thoracotomy approach of about 8 cm without rib resection in 50 patients.

A smaller length of incision translates into a reduced postoperative pain because of less tissue trauma.

Though the mean bypasss time and aortic cross clamp time were slightly higher in the study group, the difference was not statistically significant. Similar results were obtained by Benetti F and colleagues and (5, 6). However, Andres M and colleagues reported that median aortic cross clamp time (85 vs. 56 minutes) and mean bypass time (132 vs. 86 minutes) were statistically longer in the thoracotomy group (7). The difference in the results might be explained by the fact that Andres and his colleagues compared the two approaches in the patients who had already been once operated via median sternotomy approach.

Although the mean operating time was slightly higher in the patients who underwent AVR through right anterolateral thoracotomy, the difference was statistically insignificant (P=0.965). Elfriede Ruttmann and colleagues (8) reported significantly longer operating times with MIAVR through right anterior mini­thoracotomy. This difference might be accounted by a better expertise available at our centre. AVR through right anterolateral thoracotomy is utilized only at a few selected heart surgery centers at present although this approach has been standardized for mitral valve replacement. The results of AVR through right anterolateral thoracotomy are excellent only at the hands of those who have already mastered mitral valve replacement through this approach.

Although the mean ICU stay was slightly higher in the patients who underwent AVR through right anterolateral thoracotomy, the difference was not statistically significant (P=0.394). Yakub MA and colleagues (6) reported aortic valve surgeries through right anterolateral thoracotomy with almost comparable data (ICU stay=27±9 hrs). Sansone F et al. (9) also reported a comparable duration of postoperative ICU stay for the two groups.

According to the results obtained from NRS, the average pain score was higher among the patients who underwent AVR through median sternotomy compared to the study group. This assessment was done at 24, 48, and 72 hours after extubation. The results revealed a significant difference between the two groups regarding pain at all the three occasions, with median sternotomy approach being more painful. Federico J. Benetti and colleagues (4) and Benetti F et al. (5) also reported right anterolateral thoracotomy as a less painful approach. As reported by Thomas Walther and colleagues (10), early ambulation can be achieved in the patients undergoing cardiac surgeries through minimally invasive lateral minithoracotomy.

In this study, a significant difference was observed between the two groups regarding the post­operative length of hospital stay (8.0±1.4 days vs. 6.9±1.0 days; P=0.013) ger duration of post­operative hospital stay in the patients who underwent AVR through median sternotomy could be explained by the greater rate of wound infection because of which, the patients had to stay in the hospital for a longer period of time. P. N. Rao and A. S. Kumar (11) discharged the patients who underwent AVR through right anterolateral thoracotomy on the 7th post­operative day. In the same line, Benetti F and colleagues (5) performed aortic valve surgery through a right anterior minithoracotomy with a mean post­operative hospital stay of 7.7 days (4­11 days). Mattia Glauber and colleagues also (12) reported that the patients who underwent AVR through right anterolateral thoracotomy were fit to be discharged by a week.

Although the rate of wound infection was higher in the patients who underwent AVR through median sternotomy, the difference was not statistically significant (P=0.598). Andre Plass and colleagues (13) reported no wound infection (0%) in the patients who underwent AVR through right thoracotomy. According to the previous studies (14), superficial problems occurred in 1.1–6.7%, whereas the incidence of deep sternal wound complications ranged from 0.1 to 3.7% in the patients who underwent cardiac surgery through median sternotomy. Compared to the western data, the rate of infection was slightly higher in both study groups, probably owing to the overall sanitation in developing countries.

None of our patients in either group had wound dehiscence. A. Harjula and colleagues (15) reported a sternal dehiscence rate of 0.56% in their patients. The absence of sternal dehiscence in our patients may be accounted by the small sample size of the study.

In this study, 43.33% of the patients who underwent AVR through median sternotomy compared to 6.7% of the patients in the study group developed scar complications and the difference was statistically significant (P=0.035). The exact data to compare these results were not available in the literature.

In the present study, only 50% of the patients who underwent AVR through median sternotomy in comparison to 73.3% of the patients in the study group were satisfied with the cosmesis. Compared to the median sternotomy group, a greater number of patients were satisfied with the cosmesis in the study group. The difference, however, was not statistically significant (P=0.433). Yakub MA and colleagues (6) and P. N. Rao and A. S. Kumar (11) also concluded that right anterolateral thoracotomy yields cosmetically more appealing results compared to median sternotomy. The cosmetic appearance of this approach particularly benefits the female patients in whom, the scar remains hidden in the inframammary fold.

## 5. Conclusion

The right anterolateral thoracotomy approach for AVR proved to be easy to perform whilst maintaining the maximum security for the patients. Besides its better cosmetic results especially in female patients, this approach proved to have several advantages. It allows an optimal exposure of the aortic root, the aortic valve, the right atrium, and the right superior pulmonary vein, thus allowing appropriate access to all sites of cannulation. Yet, the added advantage of totally eradicating the risk of deep sternal infection is of great value. The shorter length of hospital stay and thus the cost effectiveness of this approach is also an additional relief to the family. However, it is necessary to carry out more prospective studies with larger number of patients in order to make definitive conclusions.
